# Persistence of Breakage in Specific Chromosome Bands 6 Years after Acute Exposure to Oil

**DOI:** 10.1371/journal.pone.0159404

**Published:** 2016-08-01

**Authors:** Alexandra Francés, Kristin Hildur, Joan Albert Barberà, Gema Rodríguez-Trigo, Jan-Paul Zock, Jesús Giraldo, Gemma Monyarch, Emma Rodriguez-Rodriguez, Fernanda de Castro Reis, Ana Souto, Federico P. Gómez, Francisco Pozo-Rodríguez, Cristina Templado, Carme Fuster

**Affiliations:** 1 Unitat de Biologia Cel·lular i Genètica Mèdica, Facultat de Medicina, Universitat Autònoma de Barcelona (UAB), Bellaterra, Spain; 2 CIBER Enfermedades Respiratorias (CIBERES), Bunyola, Mallorca, Spain; 3 Departament de Medicina Respiratòria, Hospital Clínic-Institut d’Investigacions Biomèdiques August Pi i Sunyer (IDIBAPS), Barcelona, Spain; 4 Departamento de Medicina Respiratoria, Hospital Clínico San Carlos, Madrid, Spain; 5 Centre de Recerca en Epidemiologia Ambiental (CREAL), Barcelona, Spain; 6 Institut Recerca Medica, Hospital del Mar, Barcelona, Spain; 7 CIBER Epidemiologia i Salut Pública (CIBERESP), Barcelona, Spain; 8 Institut de Neurociències and Unitat de Bioestadística, Facultat de Medicina, UAB, Bellaterra, Spain; 9 Departamento de Medicina Respiratoria, Complexo Hospitalario Universitario A Coruña, A Coruña, Spain; 10 Departamento de Medicina Respiratoria, Unidad Epidemiologia Clínica, Hospital 12 de Octubre, Madrid, Spain; ENEA, ITALY

## Abstract

**Background:**

The identification of breakpoints involved in chromosomal damage could help to detect genes involved in genetic disorders, most notably cancer. Until now, only one published study, carried out by our group, has identified chromosome bands affected by exposure to oil from an oil spill. In that study, which was performed two years after the initial oil exposure in individuals who had participated in clean-up tasks following the wreck of the Prestige, three chromosomal bands (2q21, 3q27, 5q31) were found to be especially prone to breakage. A recent follow-up study, performed on the same individuals, revealed that the genotoxic damage had persisted six years after oil exposure.

**Objectives:**

To determine whether there exist chromosome bands which are especially prone to breakages and to know if there is some correlation with those detected in the previous study. In addition, to investigate if the DNA repair problems detected previously persist in the present study.

**Design:**

Follow-up study performed six years after the Prestige oil spill.

**Setting:**

Fishermen cooperatives in coastal villages.

**Participants:**

Fishermen highly exposed to oil spill who participated in previous genotoxic study six years after the oil.

**Measurements:**

Chromosome damage in peripheral lymphocytes. For accurate identification of the breakpoints involved in chromosome damage of circulating lymphocytes, a sequential stain/G-banding technique was employed. To determine the most break-prone chromosome bands, two statistical methods, the Fragile Site Multinomial and the chi-square tests (where the bands were corrected by their length) were used. To compare the chromosome lesions, structural chromosome alterations and gaps/breaks between two groups of individuals we used the GEE test which takes into account a possible within-individual correlation. Dysfunctions in DNA repair mechanisms, expressed as chromosome damage, were assessed in cultures with aphidicolin by the GEE test.

**Results:**

Cytogenetic analyses were performed in 47 exposed individuals. A total of 251 breakpoints in exposed individuals) were identified, showing a non-uniform distribution in the human ideogram. Ten chromosome bands were found to be especially prone to breakage through both statistical methods. By comparing these bands with those observed in certain exposed individuals who had already participated the previous study, it was found in both studies that four bands (2q21, 3q27, 5q31 and 17p11.2) are particularly sensitive to breakage. Additionally, the dysfunction in DNA repair mechanisms was not significantly higher in oil-exposed individuals than in non-exposed individuals.

**Limitations:**

The sample size and the possibility of some kind of selection bias should be considered. Genotoxic results cannot be extrapolated to the high number of individuals who participated occasionally in clean-up tasks.

**Conclusion:**

Our findings show the existence of at least four target bands (2q21, 3q27, 5q31 and 17p11.2) with a greater propensity to break over time after an acute exposure to oil. The breaks in these bands, which are commonly involved in hematological cancer, may explain the increase of cancer risk reported in chronically benzene-exposed individuals. In addition, a more efficiency of the DNA repair mechanisms has been detected six years after in fishermen who were highly exposed to the oil spill. To date, only this study, performed by our group on the previous and present genotoxic effects, has analyzed the chromosomal regions affected by breakage after an acute oil exposure.

## Introduction

Crude oil contains a number of organic compounds, in particular benzene and highly concentrated polycyclic aromatic hydrocarbons, which can cause biological toxicity. Currently, little is known about the repercussions of oil exposure on human health after an oil tanker spill (reviewed in [1–3]). Due to some volatile organic oil compounds being carcinogenic in humans [4], it is important to determine whether there is an association between the exposure to oil and genotoxic effects during exposure as well as after a short-term (less than twelve months) or long-term (more than one year) period following exposure. To date, few human genotoxic studies of acute oil exposure have been reported, with most of them carried out during or shortly after exposure [5–12]. Long-term studies have been performed two [13–15], six [16] and seven [17] years after exposure. Most of these studies were conducted on populations exposed to the clean-up of the oil spill from the *Prestige* wreck [7–17].

In all these studies, different biomarkers such as micronuclei, comet- and chromosome-alterations assays were used. Chromosomal damage is one of the most suitable biomarkers to detect genotoxic effects, both for early and late effects, and in addition presents the advantage of being able to predict an increase in cancer risk [18–21]. Until now, relatively few studies have used chromosomal damage as a biomarker to investigate genotoxic effects in individuals who had participated in oil clean-up tasks. In all these studies an increase in chromosome damage was detected during [5], as well as two [13–15] and six years after [16] acute oil exposure.

It is known that a precise identification of the breakpoints involved in chromosome damage helps to more closely identify those genes responsible for genetic disorders, including cancer. For this reason, our group carried out a study to determine the potential existence of chromosome bands especially affected by breakages two years after the Prestige oil exposure: P2y study [14] In that work, the 2q21, 3q27 and 5q31 chromosome bands, which are commonly involved in hematological cancer, were found to be the most sensitive to breakage. A follow-up study performed six years after the exposure to oil, P6y study, revealed that the genotoxic damage persisted, suggesting that the cells of the bone marrow had been affected [16].

The aim of the present work is to determine if there exist chromosome bands especially affected by the genotoxic damage detected in the P6y study [16] and to identify if there is any correlation with those detected in P2y study in the same individuals [14]. In addition, we also aim to determine if the dysfunctions in DNA repair mechanisms found in P2y study [14] persist six years later.

## Methods

### Study population

**Fig 1:**
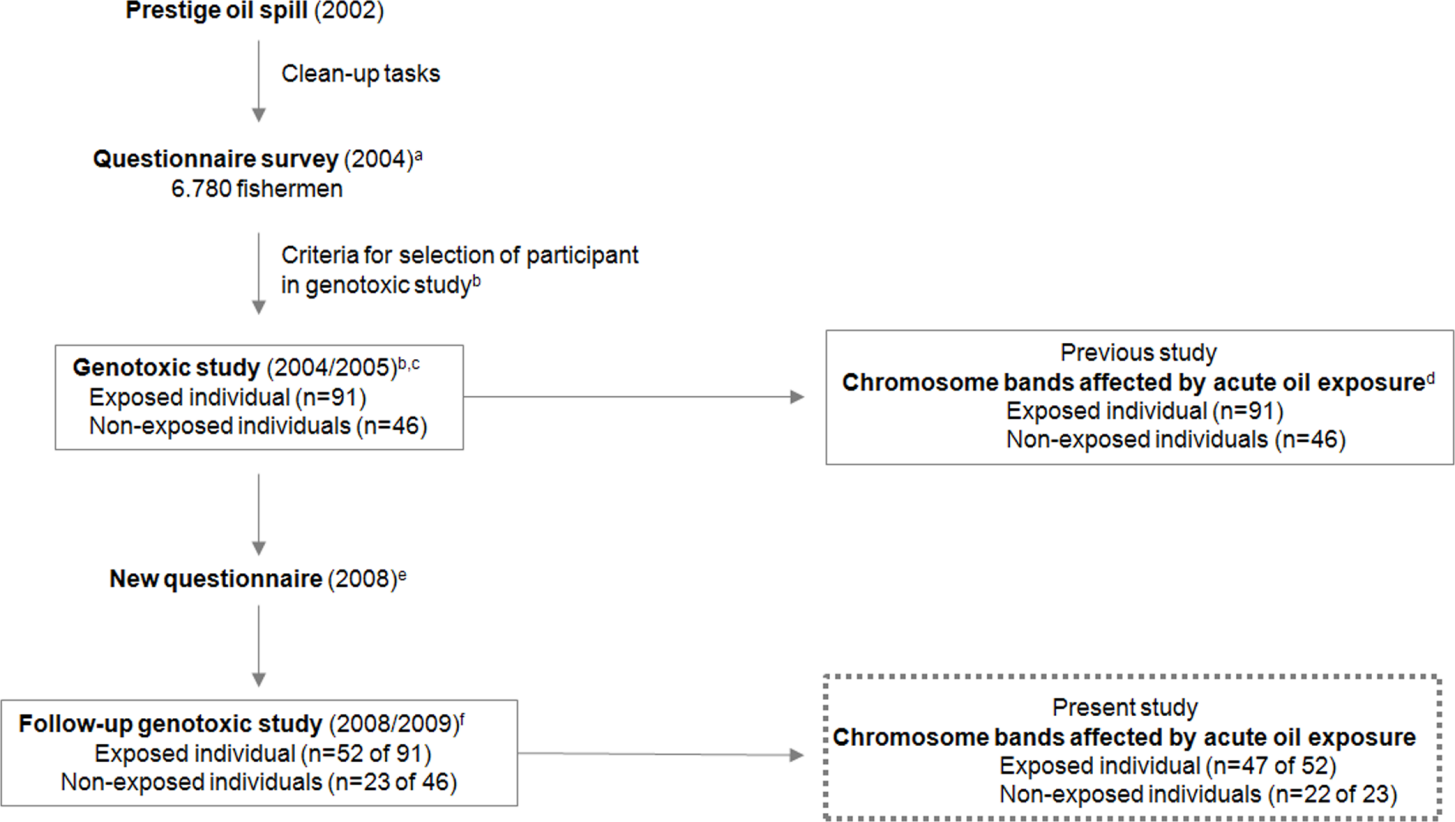
Flow diagram of the study. ^a^Detailed description in 22, ^b^Detailed description in 13, ^c^Detailed description in15, ^d^Detailed description in14, ^e^Detailed description in 23, ^f^Detailed description in16.

The present study is part of a genotoxic project in relation to Prestige oil spill (Fig 1). Briefly summarized, the oil tanker Prestige foundered near the northwestern coast of Spain in 2002, releasing a sizable quantity oil into the surrounding area. During the following months more than 300,000 persons were involved in clean-up activities. To evaluate the health effects of the oil exposure a questionnaire survey was performed which included 6,780 fishermen [22]. Only fishermen were included in our study in order to minimize other occupational sources that could act as confounders in the subsequent statistical analysis. The selection criteria of participants for the genotoxic study were established using the information included in the questionnaire, detailed in a previous report [13]. In brief, the exposed individuals were fishermen who collaborated with cleaning-up tasks (>15 days, at least four hours per day) when oil exposure was greatest. Non-exposed fishermen were those who did not participate in cleaning task for reasons other than those related to health. All individuals were local residents, non-smokers (current smokers and ex-smokers were excluded), fertile, without a history of cancer and with similar occupational conditions. Genotoxic studies, in 137 fishermen, were carried out two years after the oil spill [13,15], which included the posterior determination of chromosome bands most affected by oil exposure as determined in the P2y study [14]. A follow-up study, in 75 of the 137 individuals who participated in the previous genotoxic study, was realized four years later, that is to say, six years after the oil spill; P6y study [16]. In the present study, the health questionnaire previously used was conducted in all individuals in order to detect potential health problems during the last four years [23]. The aim of our study was an accurate localization of chromosome breakpoints in metaphases with a high quality G banding pattern. For this reason, six individuals analyzed by Hildur et al. (2015) were excluding in the present work.

The collection of the samples was carried out between November 2008 and April 2009 (between six and six-and-a-half years after the oil spill).

The present study was approved by the Ethics Committee on Clinical Research of Galicia and all participants signed the participating consent form.

### Cytogenetic analysis

Peripheral lymphocytes were cultured in RPMI-1640 medium (GIBCO Invitrogen Cell Culture, Invitrogen, Carlsbad, California) supplemented with 20% fetal bovine serum and phytohemagglutinin at 37°C. Lymphocyte culture harvesting and metaphase spreads were prepared according to standard cytogenetic protocols. Only high-quality metaphases were studied.

#### Standard lymphocyte culture

Was used to determine the chromosome bands more affected by breakages. Spontaneous chromosome damage included structural chromosome alterations evaluation (deletion, translocation, acentric, marker chromosome, etc) and chromosome lesions (gaps and breaks). At least 50 karyotypes for each individual were analyzed in order to detect structural chromosome alterations. For chromosome lesions analyses, slides were uniformly stained with Leishman. For each individual, at least 200 metaphases were investigated. The same slides were de-stained and later a G-banding technique, using Wright staining, was applied to identify the breakpoints involved in chromosome damage (chromosome lesions and alterations).

#### Lymphocyte culture with aphidicolin

Was realized to study of DNA repair efficiency in a subgroup of 18 randomly selected women because females subjects were more prevalent than males in both exposed and non-exposed individuals. Peripheral lymphocytes, obtained in the same extraction employed for chromosome damage analyses [16], were cultured in RPMI-1640 medium at 37°C for 96h. Aphidicolin (Sigma Aldrich), an inhibitor of DNA polymerase α and other polymerases, was added to the cultures 24h before harvesting at a final concentration of 0.2μM. Chromosome preparations were uniformly stained with Leishman (1:4 in Leishman buffer) allowing for the detection of induced chromosomal damage, expressed mainly as chromosomal lesions (gaps and breaks). Moreover, apparent structural chromosome alterations (rings, marker chromosomes, dicentric translocations, etc.) were also detected. A posterior G-banding technique was applied to identify the breakpoints involved in the chromosome damage. A minimum of 100 metaphases were analyzed in each participant according to conventional criteria.

All slides were coded before cytogenetic analysis so that information identifying whether individuals were exposed or non-exposed was not available until the completion of data collection.

Chromosome lesions and chromosome structural alterations were classified according to the International System for Human Cytogenetic Nomenclature [24].

### Statistical analysis

To identify the chromosomal bands most involved in breakage two statistical methods were used. First, the Fragile Site Multinomial method, FSM version 995 [14,25], was specifically used to determine chromosomal regions with a greater sensitivity to breakage. Second, a chi-square analysis was performed to test the null hypothesis of a uniform distribution among the chromosomal bands, where the bands were corrected by their length [14]. The relative length of the affected bands in relation to total genome was estimated using the diagram of the standardized human karyotype [24]. To compare the total of chromosome lesions, structural chromosome alterations and gaps/breaks between the exposed and non-exposed individuals, a generalized estimating equation, GEE, was used [14,26]. Differing levels of dysfunctions in DNA repair mechanisms, expressed as chromosome damage, between two groups of individuals were also assessed in cultures with aphidicolin by the GEE test. The GEE approach is an extension of generalized linear models designed to account for repeated, within-individual measurements. This technique is particularly indicated for when the normality assumption is not reasonable, as happens, for instance, with discrete data. The GEE model was used instead of the classic Fisher exact test because the former takes into account the possible within-individual correlation, whereas the latter assumes that all observations are independent. Since several metaphases were analyzed per individual, the GEE model is more appropriate. Statistical significance was set at p< 0.05. Statistical analyses were carried out with SAS/STAT release 9.02 (SAS Institute Inc; Cary, NC). The GEE model was fitted using the REPEATED statement in the GENMOD procedure. The conservative Type 3 statistics score was used for the analysis of the effects in the model.

## Results

### Chromosomal bands most affected by oil exposure

**Fig 2:**
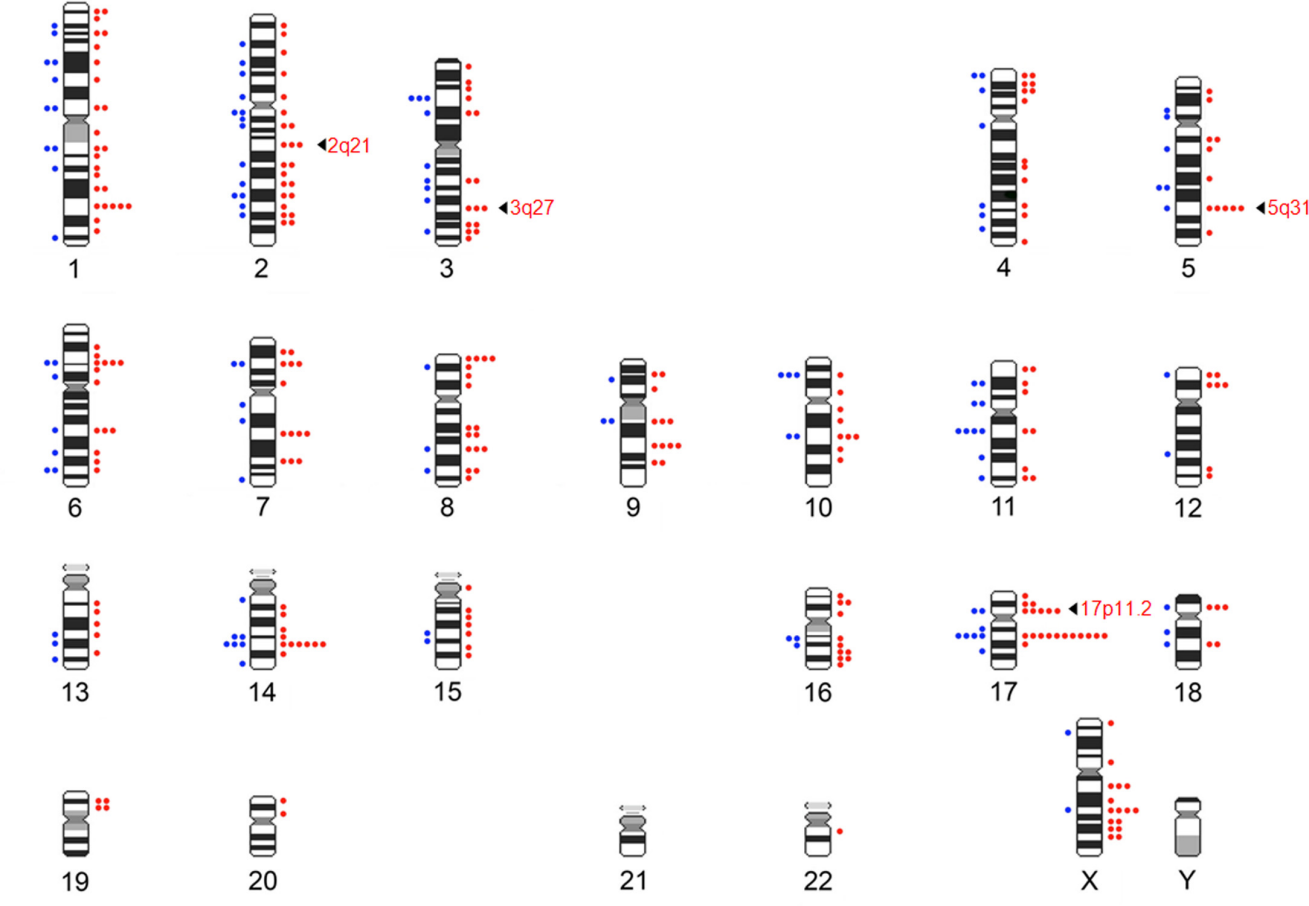
Distribution of breakpoints observed in lymphocytes metaphases from exposed to oil (right) and non-exposed (left) individuals in the human ideogram (400-band resolution). The most affected bands only in exposed are indicated by arrows.

The present study includes 69 of the 75 individuals of the previous P6y study. A total of 14,324 uniformly stained metaphases and 3,914 karyotypes were analyzed. Uniform G-banding sequential analysis allowed the identification of a total of 361 breakpoints involved in spontaneous chromosomal lesions and structural chromosome alterations in exposed and non-exposed individuals. Distribution of breakpoints in the human ideogram (at the 400-bands resolution level) was not uniform in either group of individuals as shown in Fig 2. With the exception of chromosomes 21 and Y all chromosomes were affected by damage in exposed individuals.

The chromosome bands most prone to breakages, as detected by two statistical methods in exposed and non-exposed individuals, are shown in Table 1. With the FSM method, the number of breaks required to consider a band as being non-randomly affected was ≥ 4 for exposed and ≥ 3 for non-exposed individuals. Ten bands were identified as most affected in exposed individuals vs. five in non-exposed individuals, and two of them were common to both groups. The second statistical method, using the chi-square test and taking into account the relative length of chromosomal bands, detected a higher number of bands especially prone to breakage in exposed and non-exposed individuals (35 and 28, respectively), with seven of them being present in both individual groups). The bands especially prone to breakage identified by FSM method were also detected using the second statistical method. Eight breakage-prone chromosome bands were only detected in exposed individuals (1q32, 5q31, 6p21.2, 7q22, 8p23, 9q22, 17p11.2 and Xq22) using the two statistical methods. In addition three breakage-prone chromosome bands in non-exposed (3p21, 10p13 and 11q13), and two in both groups of individuals (14q24 and 17q21), were also observed by both statistical methods. Moreover, 2p23 and 2q23 chromosome bands are only detected as more affected in exposed individuals by the two statistical methods.

**Table 1:** Chromosome bands most affected by spontaneous breakage in exposed and non-exposed individuals six years after oil exposure.

	Exposed individuals	Non-Exposed individuals
**Individuals**	47	22
**Total breakpoints identified by G-banding**	251	110
Chromosome lesions	149	63
Structural chromosome alterations	102	47
**Statistical methods used**		
Fragile site multinomial (FSM method)	**1q32**(5), **5q31**(5), **6p21.2** (4), **7q22**(4), **8p23**(4), **9q22**(4), 14q24(6), **17p11.2**(5), 17q21(11), **Xq22** (4)	3p21 (3), 10p13(3), 11q13(4), 14q24 (3), 17q21(4)
Statistical method using relative length of chromosome bands	1q22(1), **1q32**(5), 2q13(2), 2q21(3), 2q23(2), 2q34(2), 2q35(2), 3q25(3), 3q27(3), 4p15.2(2), **5q31**(5), 6p11.2(1), **6p21.2**(4), 6q21(3), 7p15(3), **7q22**(4), 7q32(3), **8p23**(4), 8q21.2(2), 9p22(2), 9q13(3), **9q22**(4), 9q32(2), 12p12(3), 12p13.1(2), 14q24(6), 16p13.1(2), **17p11.2**(5), 17p12(2), 17q21(11), 18p11.2(3), 19p13.2(2), Xq13(3), **Xq22**(4), Xq24(2)	1p13(2), 1q21(2), 2p14(1), 2q11.2(2), 2q32(2), 3p21(3), 3q13.2(1), 4p16(2), 4q31.3(1), 4q33(1), 5p12(1), 5q22(2), 6p21.2(2), 6q25(2), 7p15(2), 9q13(2), 10p13(3), 10q22(2), 11p11((2), 11p14(2), 11q13(4), 12p13.1(1), 14q24(3), 15q23(1),16q13(2), 17p11.2(2), 17q12(1), 17q21(4)

Numbers in parentheses indicate the breaks located in chromosome band. Bold chromosome bands are present only in exposed individuals using two statistical methods.

To compare these chromosome bands most affected by breakage with those observed the previous study [14], it was found that four bands (2q21, 3q27, 5q31 and 17p11.2) are particularly sensitive to breakage in both studies (Table 2) in different oil exposed participants (Table 3).

**Table 2:** Chromosome bands most affected by spontaneous breakage only in exposed individuals two and six years after oil spill exposure.

	Cytogenetic study six years after oil exposure (Present P6y study)	Cytogenetic study two years after oil exposure (Previous P2y study)
**Exposed individuals**	47	91
**Total breakpoints identified by G-banding**	251	203
**Statistical methods used**		
Fragile site multinomial (FSM method)	1q32, **5q31**, 6p21.2, 7q22, 8p23, 9q22, **17p11.2**, Xq22	**2q21**, **3q27, 5q31**
Statistical method using relative length of chromosome bands	1q22, 1q32, 2q13, **2q21,** 2q23, 2q34, 2q35, 3q25, **3q27**, 4p15.2, **5q31**, 6p11.2, 6q21, 7q22, 7q32, 8p23, 8q21.2, 9p22, 9q22, 9q32, 12p12, 16p13.1, **17p11.2,** 18p11.2, 19p13.2, Xq13, Xq22, Xq24	1p34.1, **2q21, 3q27,** 4q33, 12q11, 13q11, **17p11.2**, 18q11.2

Bold chromosome bands are present only in exposed individuals in two studies.

**Table 3:** Type of spontaneous chromosome damage on the most affected bands in exposed individuals two and six years after oil spill exposure.

Chromosome Band	Present P6y study Type of chromosome damage (Participant No.)	Previous P2y study Type of chromosome damage (Participant No.)
2q21	chtg (E69); chsg (E78); del+ace (E49)	chtg (E1); chsg (E20); t(2;8)(q21;p22) (E41); chtg (E65)
3q27	chtg (E56); chtg (E88); chsb (E88)	chsg (E24); chtb (E11); chtg (E51); chsb (E72)
5q31	chtg (E74); del (E60); chtg(E24); chsg (E49); chtg (E20)	chtg (E4); t(5;16)(q31;p13.1) (E36); t(5;16)(q31;p13.1) (E36); chtb (E77)
17p11.2	del (E15); del (E85); del (E49); del (E37); del (E84)	chsb (E7); del (E61); del (E90)

ace, acentric; chrb, chromosome break; chrg, chromosome break; chtb, chromatid break; chtg, chromatid gap; del, deletion; fragment; t, reciprocal translocation.

### DNA repair efficiency

Chromosome lesions induced by aphidicolin were studied in a subgroup of 18 individuals. A higher number of induced lesions was observed in exposed (40,1%) vs. non-exposed individuals (30,4%), but the difference was not statistically significant (337 lesions from 840 uniform stain metaphases in exposed vs. 319 from 1,050 non-exposed individuals; p = 0.1258).

A total of 357 breakpoints were accurately identified. Bands most affected by breakage, identified by the same statistical methods, are shown in Table 4. Eight chromosome bands especially affected by breakage were detected in exposed individuals using both statistical methods. Chromosome bands 3p14, 6q26, 16q23, 17q21 and Xp22 were especially prone to damage in exposed and non-exposed individuals in the two statistical methods used. Only 2p23 and 2q32 bands were found in exposed group using both statistical methods.

**Table 4:** Chromosomal damage induced by aphidicolin in exposed and non-exposed individuals to oil six years after oil exposure.

	Exposed individuals	Non-Exposed individuals
**Individuals**	8	10
**Total breakpoints identified by G-banding**	196	161
Gaps	137	113
Breaks	59	48
**Statistical methods used**		
Fragile site multinomial (FSM method)	2p23(5), 2q32(5), 3p14(38), 6q26(7), 11q13(5), 16q23(30), 17q21(17), Xp22.2(9)	3p14(29), 6q26(19), 11q13(4), 16q23(38), 17q21(8), Xp22.2(4)
Statistical method using relative length of chromosome bands	2p23(5), 2q32(5), 3p14(38), 6p21.2(2), 6q21(4), 6q26(7), 7q32(4), 11q13(5), 16q23(30), 17q21(17), Xp22.2(9)	3p14(29), 6p21.3(3), 6q26(19), 7q32(3), 16q23(38), 17q21(8), Xp22.2(4)

Numbers in parentheses indicate the number of breaks located in chromosome band.

## Discussion

Until now, although the human genome has been widely studied, little is known about the specific genome regions affected by oil exposure. The present study was included in a more extended follow-up project on the Prestige oil spill designed to determine if chromosome damage persisted. This follow-up project is the first reported in the literature to use biomarkers to test chromosome alterations. Two studies [13,16], carried out previously by our group, two and six years after oil spill exposure, suggested that the stem cells of the bone marrow could have been affected. The aim of the present study was the identification of specific chromosome bands especially prone to breakage after an acute oil exposure and the relation of these bands with possible genes involved in cancer.

### Chromosomal bands most affected by oil exposure

It is known that a chromosome damage biomarker is useful for short and long-term evaluation after a genotoxic exposure and moreover it is able to predict an increase in the risk of cancer [18–21]. In addition, this biomarker allows for the identification of chromosome breakpoints in the genome and the chromosomal bands most prone to breakage. In previous and present genotoxic analysis ([14] and present study), a non-uniform distribution of breakpoints induced by oil exposure in the human ideogram was found. Moreover, a notable correlation between breakpoints and fragile-site regions (up to 84%, according to Mrasek et al. [27]) was observed. These findings were relevant because fragile sites are large chromosome regions, over megabases, prone to breakage upon replication stress and are a driving force of oncogenesis [28,29]; suggesting that breakpoints originating from oil exposure may affect the genome region which themselves are prone to breakage leading to chromosome instability and the earliest stages of cancer development. Moreover, our findings show that four chromosome bands: 2q21, 3q27, 5q31 and 17p11.2, which were especially affected by acute oil exposure, were detected only in exposed individuals in both studies ([14] and present study), discarding factors involved in lymphocyte cultures. All these bands correspond to regions where fragile sites are located, according to human genome browsers including CNBI (http://www.ncbi.nlm.nih.gov/): FRA2F (2q21.3; aphidicolin-type, common), FRA3C (3q27; aphidicolin-type, common), FRA5C (5q31.1; aphidicolin-type, common), and FRA17A (17p11.2; distamycin A type, rare). Although not all fragile sites may be equally involved in cancer development [28,30], the correlation between breaks induced by oil exposure and fragile sites may suggest some relation to cancer development.

To determine whether any of the genes located in these four bands could explain the cellular disorders involved in cancer, we searched the information available from genome browsers like NCBI (www.ncbi.nlm.nih.gov/). In the 2q21 band genes are located which relate to the cellular cycle control (CCNT2, MAP3K19 and MZT2A), DNA replication/repair mechanisms (ERCC3, MCM6), proto-oncogene (MIR28 and RAB6C) and tumor suppressor genes (CXCR4 and NMTC1). In the 3q27 band are found the THPO gene, involved in the cellular development process, and the BCL2 tumor suppressor gene. In the 5q31 band genes have been observed which are related with the cellular cycle control (CDC25C, CSF2, FGF1 and GDF9S), DNA replication/repair mechanisms (RAD50), regulator chromatin (HDAC3 and SKP1), proto-oncogenes (TGFBI), and tumor suppressor genes (EGR1, IRF1 and HINT1). And finally, in 17p11.2 studies have identified the MAPK7 gene (mitogen activity), TOP3A gene (DNA replication/repair mechanisms), and TNFRSF13 gene (tumor necrosis factor receptor family). In addition to the genes related to cancer located in these bands, it is interesting to note that: (i) acute oil exposure could affect the stem cells of the bone marrow leading to genomic instability and an increased risk of hematopoietic malignancies, such as suggested previously by Hildur et al. [16]; (ii) individuals chronically exposed to benzene have a higher risk of cancer [31, 32]; and (iii) a significant number of chromosome alterations in hematological diseases, such as patients with T-cell lymphoma, acute lymphoblastic leukemia and acute myeloid leukemia, are associated with these four bands (reviewed by [14,33,34]). For all these reasons, we have suggested that acute oil exposure may be involved in the formation of cancer, particularly hematological, by causing chromosomal damage in the same way that chronic benzene exposure does. Since none of individuals included in the present study had developed cancer, a more extended follow-up study [23] realized by our group of 622 individuals revealed seven who had developed cancer (skin, prostate, colon, bladder, uterine and breast), six of them had been exposed to oil from the spill (unpublished data). We believe that it is still too early to assess the acute oil effect on cancer; but health authorities in the region have been alerted. Our findings reveal the need to realize additional genetic studies to clarify if there is an association between acute oil exposure and an increased risk of cancer.

Cytogenetic studies in individuals chronically exposed to oil or its compounds, mainly benzene, have reported that the chromosome 2 centromere, 4q21 and 7q22 bands and reciprocal translocation t(8;21) were especially affected [35,36]. All these chronic exposure studies were performed using G banding analysis, and the same analysis was employed in the present study. The loss of chromosomes 5 and 7, deletions of 5q31 and 7q22, and translocations of t(9;22), t(15;17), t(8;21) and t(14;18) have also been detected using the FISH technique [37–40].

It is worth clarifying that conventional G-banding allows for the identification of breakpoints involved in chromosome damage without focusing on specific chromosomes beforehand as opposed to the FISH methodology which detects only chromosome damage in default specific chromosomes and presents serious difficulties in identifying the breakpoints. Chromosome band 5q31 is the only band described as especially sensitive to oil in chronic [37] and in accidental but acute exposure ([14] and present study). Moreover, a significant amount of the chromosome reorganization in hematopoietic pathologies involves the 5q31 band in patients with acute lymphoblastic leukemia, myelodysplastic syndrome, chronic myelomonocytic leukemia and acute myeloid leukemia (revised in [14,33,34]), which supports the possible existence of an association between acute/chronic oil exposure and increased cancer risk, particularly hematological.

Three chromosome bands (3p21, 10p13 and 11q13) were found only in the non-exposed group using both statistical methods. It seems unlikely that acute exposure to oil prevents damage of these bands, rather it may be due to indirect exposure to the much slower and less intense oil (the non-exposed group is not a true control group, but a fishermen group who did not participate in cleaning task).

In the present study, an unexpectedly high number of breaks in the 14q24 and 17q21 bands were detected in individuals both exposed and non-exposed to the oil spill. The presence of breaks detected in non-exposed individual could be explained by an indirect exposure to some oil compounds during the years following the spill, such has been suggested by Hildur et al. [16]. This finding indicates that non-exposed individuals residing in proximity to an oil spill should also be monitored due a probable increased risk of cancer in relation to the general population. The present hypothesis is supported by the increase of cancer, especially leukemia, in people living in close proximity to oil refineries [41,42] and in proximity to petroleum storage tanks [43].

However, the clinical relevance of accidental oil exposure and its association with an increased risk of cancer (assessed by breakage in these target bands) must be confirmed by further follow-up genotoxic studies in order to detect cancer early in individuals exposed.

### DNA repair efficiency

It has been reported that the chromosome lesions produced in the presence of an inhibitor of DNA polymerase are indicative of the replication stress (by aphidicolin) and break repair efficiency [44,45]. In the present study we performed a replication stress/DNA repair study, using cultures with aphidicolin, in order to compare the chromosome damage observed with that of the previous P2y study [14]. In this P2y study dysfunction in the replication stress/DNA repair was observed in exposed individuals two years after the oil spill. The present P6y study, carried out in the same individuals, shows no differences between exposed and non-exposed, indicating that the replication/DNA repair have become efficient again after 6 years of an acute exposure. This fact may be explain the lower spontaneous chromosome damage in standard culture lymphocytes detected in exposed individuals in follow-up P6y study [16] vs. P2y study [14]. In opposite, the individuals chronically exposed to benzene showed a low efficiency in DNA repair mechanisms, but by using ionizing radiation instead of aphidicolin [46–48]. The fact that dysfunctions in DNA repair mechanisms might predispose cells to the development of cancer [49] could explain the increased risk of cancer, reported previously in chronically benzene-exposed individuals [31, 32]. Thus, the efficiency in replication and DNA repair, detected six years after of the acute oil exposure, suggest a possible lower risk of cancer. However, present results should be taken with caution because only eight exposed individuals were analyzed. Moreover, the genotoxic marker used in present study is not able to differentiate between whether the oil-related DNA damage affected DNA repair genes or if individuals with existing DNA repair deficiency were affected preferentially.

On the other hand, in the present study a correlation between the common fragile sites most frequent in the general population [27] and the 3p14, 6q26, 7q32, 17q21, 16q23, Xp22.2 bands most affected by breakage was observed using aphidicolin. None of these bands coincide with those four target bands (2q21, 3q27, 5q31 and 17p11.2) with a greater propensity to break over time after an acute oil exposure. This findings support the hypothesis of Mrasek et al. [27] that common fragile sites are breakage-prone regions less dependent on inducing chemicals than originally supposed.

Moreover, the most affected chromosome bands in exposed individuals are 2p23 and 2q23. In these two bands are locate gens that can be involved in malignancies, such SPDYA (cell cycle regulator) and ALK (anaplastic lymphoma receptor tyrosine kinase) in 2p23 band and LYPD6B (cancer/testis antigen 116) and RIF1 *(*replication timing regulatory factor 1) in 2q23 band (www.ncbi.nlm.nih.gov/). However, more studies must be carried out, in a future, to link cancer-risk and the acute oil-exposure.

## Conclusions

To date, only previous and present genotoxic effects, performed by our group, have analyzed the chromosomal regions affected by breakage after an acute oil exposure. Our findings show the persistence of 2q21, 3q27, 5q31 and 17p11.2 chromosome bands six years after acute oil exposure, which are commonly involved in hematological cancer. The breaks in these regions may cause deletions or disruptions of functional genes would explain the increased risk of cancer in chronically benzene-exposed individuals. However, more number of individuals and sophisticated analysis must be realized to link cancer-risk and the acute oil-exposure. We wish to emphasize that our findings cannot be extrapolated to the individuals who participated occasionally in clean-up tasks nor all of the local population in the area of the oil spill because of the sample size and the very rigorous selection of individuals.

## References

[1] Aguilera F, Méndez J, Pásaro E, Laffon B. Review of the effects of exposure to spilled oils on human health. J Appl Toxicol. 2010;30:291–301. 10.1002/jat.1521 20499335

[2] Goldstein BD, Osofsky HJ, Lichtveld MY. The Gulf oil spill. N Engl J Med. 2011,364,1334–1348. 10.1056/NEJMra1007197 21470011

[3] D'Andrea MA, Reddy GK. Health risks associated with crude oil spill exposure. Am J Med. 2014;127:886.e9–13. 10.1016/j.amjmed.2014.04.035 24859637

[4] IARC (1998). International Agency for Research on Cancer. Monographs on the Overall Evaluations of Carcinogenicity: An Updating of IARC Monographs Volumes 1 to 42 IRAC Monographs Carcinogenic risk to Human (Suplement7). Lyon: International Agency for Research on Cancer.

[5] Clare MG, Yardley‐Jones A, Maclean AC, Dean BJ. Chromosome analysis from peripheral blood lymphocytes of workers after an acute exposure to benzene. Br J Ind Med. 1984;41:249‐253. 6722051 10.1136/oem.41.2.249 PMC1009291

[6] Cole J, Beare DM, Waugh AP, Capulas E, Aldridge KE, Arlett CF, et al Biomonitoring of possible human exposure to environmental genotoxic chemicals: lessons from a study following the wreck of the oil tanker Braer. Environ Mol Mutagen. 1997;30:97‐111. 9329634

[7] Laffon B, Fraga‐Iriso R, Perez‐Cadahia B, Mendez J. Genotoxicity associated to exposure to Prestige oil during autopsies and cleaning of oil‐contaminated birds. Food Chem Toxicol. 2006;44:1714‐1723. 16814914 10.1016/j.fct.2006.05.010

[8] Perez-Cadahia B, Laffon B, Pásaro E, Méndez J. Genetic damage induced by accidental environmental pollutants. Scientific World Journal 2006;6:1221–1237. 17013527 10.1100/tsw.2006.206 PMC5917237

[9] Perez‐Cadahia B, Lafuente A, Cabaleiro T, Pásaro E, Méndez J, Laffon B. Initial study on the effects of Prestige oil on human health. Environ Int. 2007;33:176‐185. 17055056 10.1016/j.envint.2006.09.006

[10] Perez‐Cadahia B, Laffon B, Porta M, Lafuente A, Cabaleiro T, López T, et al Relationship between blood concentrations of heavy metals and cytogenetic and endocrine parameters among subjects involved in cleaning coastal areas affected by the “Prestige” tanker oil spill. Chemosphere. 2008;71:447‐455. 10.1016/j.chemosphere.2007.10.053 18221981

[11] Perez‐Cadahia B, Laffon B, Valdiglesias V, Pásaro E, Méndez J. Cytogenetic effects induced by Prestige oil on human populations: The role of polymorphisms in genes involved in metabolism and DNA repair. Mutat Res. 2008;653:117‐123. 10.1016/j.mrgentox.2008.04.002 18495522

[12] Perez-Cadahía B, Méndez J, Pásaro E, Lafuente A, Cabaleiro T, Laffon B. Biomonitoring of human exposure to Prestige Oil: Effects on DNA and endocrine parameters. Environ Health Insights 2008;2:83–92. 21572833 10.4137/EHI.S954 PMC3091333

[13] Rodriguez-Trigo G, Zock JP, Pozo-Rodríguez F, Gómez FP, Monyarch G, Bouso L, et al Health changes in fishermen 2 years after clean‐up of the Prestige oil spill. Ann Intern Med. 2010;153:489–499. 10.7326/0003-4819-153-8-201010190-00279 20733177

[14] Monyarch G, de Castro Reis F, Zock JP, Giraldo J, Pozo-Rodríguez F, Espinosa A, et al Chromosomal bands affected by acute oil exposure and DNA repair errors. PLoS One 2013;8(11):e81276 10.1371/journal.pone.0081276 24303039 PMC3841120

[15] Biern G, Giraldo J, Zock JP, Monyarch G, Espinosa A, Rodríguez-Trigo G, et al Human genotoxic study carried out two years after oil exposure during the clean-up activities using two different biomarkers. J Mar Sci. Eng 2015;3:1334–1348.

[16] Hildur K, C Templado C, Zock JP, Giraldo J, Pozo-Rodríguez F, Frances A, et al Follow-up genotoxic study: Chromosome damage two and six years after exposure to the Prestige oil spill. PLoS One. 2015;10:e0132413 10.1371/journal.pone.0132413 26221948 PMC4519162

[17] Laffon B, Aguilera F, Ríos-Vázquez J, Valdiglesias V, Pásaro E. Follow-up study of genotoxic effects in individuals exposed to oil from the tanker Prestige, seven years after the accident. Mutat Res 2014;760:10–16. 10.1016/j.mrgentox.2013.09.013 24370900

[18] Norppa H, Bonassi S, Hansteen IL, Hagmar L, Strömberg U, Rössner P, et al Chromosomal aberrations and SCEs as biomarkers of cancer risk. Mutat Res. 2006; 600:37–45. 16814813 10.1016/j.mrfmmm.2006.05.030

[19] Au WW. Usefulness of biomarkers in population studies: from exposure to susceptibility and to prediction of cancer. Int J Hyg Environ Health. 2007;210: 239–246. 17174154 10.1016/j.ijheh.2006.11.001

[20] Bonassi S, Norppa H, Ceppi M, Strömberg U, Vermeulen R, Znaor A, et al Chromosomal aberration frequency in lymphocytes predicts the risk of cancer: results from a pooled cohort study of 22.358 subjects in 11 countries. Carcinogenesis. 2008;29:1178‐1183. 10.1093/carcin/bgn075 18356148 PMC2443275

[21] Frohling S, Dohner H. Chromosomal abnormalities in cancer. N Engl J Med. 2008;359:722–734. 10.1056/NEJMra0803109 18703475

[22] Zock JP, Rodriguez-Trigo G, Pozo-Rodriguez F, Barberà JA, Bouso L, Torralba Y, et al Prolonged respiratory symptoms in clean-up workers of the Prestige oil spill. Am J Respir Crit Care Med. 2007;176:610–616. 17556713 10.1164/rccm.200701-016OC

[23] Zock JP, Rodríguez-Trigo G, Rodríguez-Rodríguez E, Espinosa A, Pozo-Rodríguez F, Gómez F, et al Persistent respiratory symptoms in clean-up workers 5 years after the Prestige oil spill. Occup Environ Med. 2012;69:508–513. 10.1136/oemed-2011-100614 22539655

[24] ISCN 2013 An International System for Human Cytogenetic Nomenclature. Shafer LG, McGowan-Jordan J and Schmid M (eds); S. Karger. Basel 2013.

[25] Greenbaum IF, Fulton JK, White ED, Dahm PF. Minimum sample sizes for identifying chromosomal fragile sites from individuals: Monte Carlo estimation. Hum Genet. 1997;101:109‐112. 9385380 10.1007/s004390050596

[26] Liang KY, Zeger SL (1986). Longitudinal data analysis using generalized linear models. Biometrika. 73: 13–22.

[27] Mrasek K, Schoder C, Teichmann AC, Behr K, Franze B, Wilhelm K, et al Global screening and extended nomenclature for 230 aphidicolin-inducible fragile sites, including 61 yet unreported ones. Int J Onc. 2010;36:929–940 10.3892/ijo_00000572 20198338

[28] Debatisse M, Le Tallec B, Letessier A, Dutrillaux B, Brison O. Common fragile sites: mechanisms of instability revisited. Trends Genet. 2012;28:22–32. 10.1016/j.tig.2011.10.003 22094264

[29] Aqeilan RI. Role of common fragile sites and corresponding genes in cancer development. Cell Mol Life Sci. 2014;71:4487–4488. 10.1007/s00018-014-1716-y 25238781 PMC11113964

[30] Georgakilas AG, Tsantoulis P, Kotsinas A, Michalopoulos I, Townsend P, Gorgoulis VG. Are common fragile sites merely structural domains or highly organized “functional” units susceptible to oncogenic stress? Cell Mol Life Sci. 2014;71:4519–4544. 10.1007/s00018-014-1717-x 25238782 PMC4232749

[31] Aksoy M. Malignancies due to occupational exposure to benzene. Am J Ind Med. 1985;7:395–402. 4003402 10.1002/ajim.4700070506

[32] Schnatter AR, Glass DC, Tang G, Irons RD, Rushton L. Myelodysplastic syndrome and benzene exposure among petroleum workers: an international pooled analysis. J Natl Cancer Inst 2012;104:1724–1737. 10.1093/jnci/djs411 23111193 PMC3502195

[33] De Braekeleer E, Auffret R, Douet-Guilbert N, Basinko A, Le Bris MJ, Morel F, et al Recurrent translocation (10;17)(p15;q21) in acute poorly differentiated myeloid leukemia likely results in ZMYND11-MBTD1 fusion. Leuk Lymphoma 2014;55:1189–1190. 10.3109/10428194.2013.820292 23915195

[34] Douet-Guilbert N, Tous C, Le Flahec G, Bovo C, Le Bris MJ, Basinko A, et al Translocation t(2;7)(p11;q21) associated with the CDK6/IGK rearrangement is a rare but recurrent abnormality in B-cell lymphoproliferative malignancies. Cancer Genet. 2014;207:83–86. 10.1016/j.cancergen.2014.02.009 24726269

[35] Sasiadek M, Jagielski J, Smolik R. Localization of breakpoints in the karyotype of workers professionally exposed to benzene. Mutat Res. 1989;224:235–240. 2797038 10.1016/0165-1218(89)90161-4

[36] Kim SY, Choi JK, Cho YH, Chung EJ, Paek D, Chung HW. Chromosome aberrations in workers exposed to low levels of benzene: association with genetic polymorphisms. Pharmacogenetics 2004;14:453–463. 15226677 10.1097/01.fpc.0000114751.08559.7b

[37] Zhang L, Wang Y, Shang N, Smith MT. Benzene metabolites induce the loss and long arm deletions of chromosomes 5 and 7 in human lymphocytes. Leukemia Res. 1998;22:105–113. 9593466 10.1016/s0145-2126(97)00157-4

[38] Stillman WS, Varella-Garcia M, Irons RD. The benzene metabolite, hydroquinone, selectively induces 5q31—and—7 in human CD34+CD19- bone marrow cells. Experimental Hematology. 2000;28:169–176. 10706073 10.1016/s0301-472x(99)00144-7

[39] Marcon F, Zijno A, Dobrowolny G, Carere AS, Crebelli R: Detection of 1cen-1q12 lesions in different phases of cell cycle: dual colour FISH analysis of peripheral lymphocytes from subjects with occupational exposure to petroleum fuels. Mutagenesis. 2002;17:157–162. 11880545 10.1093/mutage/17.2.157

[40] Ji Z, Zhang L. Chromosomics: detection of numerical and structural alterations in all 24 human chromosomes simultaneously using a novel OctoChrome FISH assay. J Vis Exp. 2012;60 10.3791/3619.10.3791/3619 PMC3369631 22331009

[41] Barregard L, Holmberg E, Sallsten G. Leukemia incidence in people living close to an oil refinery. Environ Res. 2009;109: 985–990. 10.1016/j.envres.2009.09.001 19781695

[42] Salerno C, Berchialla P, Palin LA, Vanhaecht K, Panella M. Cancer morbidity of residents living near an oil refinery plant in North-West Italy. Int J Environ Health Res 2013;23:342–351. 10.1080/09603123.2012.733938 23067277

[43] Zusman M, Dubnov J, Barchana M, Portnov BA. Residential proximity to petroleum storage tanks and associated cancer risks: Double Kernel Density approach vs. zonal estimates. Sci Total Environ. 2012;441:265–276. 10.1016/j.scitotenv.2012.09.054 23147397

[44] Slyskova J, Naccarati A, Polakova V, Pardini B, Vodickova L, Stetina R, et al DNA damage and nucleotide excision repair capacity in healthy individuals.Environ Mol Mutagen. 2011;52:511–517. 10.1002/em.20650 21520291

[45] Speit G, Leibiger C, Kuehner S, Högel J. Further investigations on the modified comet assay for measuring aphidicolin-block nucleotide excision repair. Mutagenesis. 2013;28:145–151. 10.1093/mutage/ges063 23221037

[46] Hallberg LM, Bechtold WE, Grady J, Legator MS, Au WW. Abnormal DNA repair activities in lymphocytes of workers exposed to 1,3-butadiene. Mutat Res. 1997;383:213–221. 9164482 10.1016/s0921-8777(97)00004-9

[47] Navasumrit P, Chanvaivit S, Intarasunanont P, Arayasiri M, Lauhareungpanya N, Parnlob V, et al Environmental and occupational exposure to benzene in Thailand. Chem Biol Interact 2005;153:75–83. 15935802 10.1016/j.cbi.2005.03.010

[48] Chanvaivit S, Navasumrit P, Hunsonti P, Autrup H, Ruchirawat M. Exposure assessment of benzene in Thai workers, DNA-repair capacity and influence of genetic polymorphisms.Mutat Res. 2007;626:79–87. 17095285 10.1016/j.mrgentox.2006.09.007

[49] Fu XJ, Shi XJ, Lin K, Lin H, Huang WH, Zhang GJ, et al Environmental and DNA repair risk factors for breast cancer in South China. Int J Hyg Environ Health. 2015;218(3):313–318. 10.1016/j.ijheh.2015.01.001 25616561

